# Selecting the dosage of ceftazidime–avibactam in the perfect storm of nosocomial pneumonia

**DOI:** 10.1007/s00228-019-02804-z

**Published:** 2019-12-14

**Authors:** Shampa Das, Diansong Zhou, Wright W. Nichols, Andy Townsend, Paul Newell, Jianguo Li

**Affiliations:** 1 AstraZeneca, Mereside, Alderley Park, Macclesfield, SK10 4TG UK; 2 Antimicrobial Pharmacodynamics and Therapeutics, Department of Molecular and Clinical Pharmacology, University of Liverpool, Sherrington Building, L69 3GA Liverpool, UK; 3 AstraZeneca Pharmaceuticals LP, 35 Gatehouse Drive, Waltham, MA 02451 USA; 4 Present Address: Didsbury, Manchester, UK; 5 Present Address: Pfizer, Walton Oaks, UK; 6 Present Address: F2G Ltd., Manchester, UK; 7 Present Address: Chadds Ford, USA

**Keywords:** Ceftazidime–avibactam, Nosocomial pneumonia, Antibiotic therapy, Pharmacokinetics, Pharmacodynamics, Dosage selection

## Abstract

**Purpose:**

Ceftazidime–avibactam is a novel β-lactam/β-lactamase inhibitor combination recently approved in Europe and the USA for the treatment of adults with hospital-acquired pneumonia (HAP) and ventilator-associated pneumonia (VAP), among other indications. In the phase III REPROVE trial (NCT01808092), ceftazidime–avibactam demonstrated non-inferiority to meropenem for the treatment of patients with nosocomial pneumonia (NP), including VAP. As ceftazidime–avibactam was not studied in patients with NP prior to REPROVE, selecting an appropriate dosage regimen in the “perfect storm” of NP required careful consideration of potential determinants and confounders of response specific to the NP patient population.

**Methods:**

This review describes the series of preclinical studies and pharmacokinetic/pharmacodynamic (PK/PD) analyses that supported ceftazidime–avibactam dosage selection for patients with NP/VAP (2000/500 mg by 2-h intravenous infusion every 8 h, adjusted for renal function). In parallel, important considerations for antibiotic dosage selection in patients with NP are highlighted, including adequate drug penetration into the lungs, the suitability of murine-derived plasma PK/PD targets, evaluation of MIC distributions against clinical bacterial isolates from patients with NP, and consideration of PK in patients with NP, who are often critically ill. These analyses also supported the European approval of ceftazidime–avibactam for adults with HAP, including VAP, before the completion of REPROVE.

**Conclusions:**

This work serves as a successful practical example of dosage design for a new antibacterial drug therapy in the indication of NP, including VAP, where previous drug therapies have failed, possibly as a result of evaluation of too few variables, thereby limiting the accuracy of pharmacodynamic predictions.

**Electronic supplementary material:**

The online version of this article (10.1007/s00228-019-02804-z) contains supplementary material, which is available to authorized users.

## Introduction

Nosocomial pneumonia (NP), also referred to as hospital-acquired pneumonia (HAP), accounts for approximately 15% of hospital-acquired infections [1], and ventilator-associated pneumonia (VAP), a subgroup of NP, is among the most common infections in intensive care units [2, 3]. NP, and particularly VAP, are associated with high morbidity and mortality rates [4, 5], and in many regions, antimicrobial resistance is making these infections increasingly difficult to treat [5]. *Pseudomonas aeruginosa* and Enterobacteriaceae including *Klebsiella pneumoniae* and *Escherichia coli* are the most frequently isolated Gram-negative bacteria from hospitalized patients with pneumonia worldwide, of which many show reduced (< 90%) susceptibility to commonly used antimicrobials for pneumonia such as third-generation cephalosporins, thereby increasing reliance on carbapenems [6–8]. Unfortunately, the increasing resistance among clinically important Gram-negative bacteria in recent decades has occurred in the context of declining development of new antimicrobial therapies, particularly those targeting Gram-negative bacteria [9]: between 2005 (approval of tigecycline) and 2017 (approval of ceftazidime–avibactam), no new treatments were approved for NP/VAP due to Gram-negative bacteria.

NP represents a “perfect storm” for antimicrobial drug therapy for various reasons. First, as the infection occurs in the lungs, there is a potential for delayed or insufficient free drug penetration to the infection site. Second, there is evidence that bacteria isolated from patients with NP/VAP are less susceptible to antibacterial agents than isolates of the same species from other infections; such increased minimum inhibitory concentrations (MICs) have adversely influenced the outcomes of some clinical trials of new drugs in this population [10, 11]. Third, the reduced susceptibility of these bacteria to currently available antibiotics [6] can be compounded by the rapid elimination of renally cleared agents in some patients due to augmented renal clearance (ARC) [12]. Finally, alterations in organ function and hemodynamics in critically ill patients with NP can result in wide variability in antibiotic pharmacokinetics (PKs) and thus affect the ability to achieve therapeutic concentrations [13–15].

As highlighted by Ambrose and colleagues [10], for sponsors of new antimicrobial candidate therapies for NP/VAP, careful consideration of PK/pharmacodynamic (PD) determinants and confounders of response is required to ensure appropriate dosage regimen selection before starting clinical trials. The pitfalls of developing drugs for use in pneumonia without accounting for the above factors have been demonstrated for other antimicrobial therapies. The decreased in vitro activity of daptomycin against Gram-positive bacteria in the presence of pulmonary surfactant was suggested as a possible explanation for its failure in clinical trials for community-acquired pneumonia [16]. Initial preclinical studies of ceftobiprole assessed penetration of the drug into mouse but not human epithelial lining fluid (ELF), the fluid layer covering the mucosae of the alveoli and of the small and large airways [17]. The median ceftobiprole area under the curve (AUC) ratio for ELF/plasma was 0.69 in mice, whereas the ratio in humans was subsequently found to be only 0.15, resulting in an underestimation of the effective dosage required for phase III trials in patients with NP. Moreover, failure to consider ARC when determining dosages may also result in the failure of clinical trials. For example, in trials of doripenem, tigecycline, and ceftobiprole, ARC was implicated as a factor in their relatively low efficacy [18].

In this narrative review, we describe how the ceftazidime–avibactam dosage regimen was selected for the treatment of patients with NP, including VAP, and highlight important considerations for selecting antibiotic dosage regimens for this indication. Reviewing these considerations may be instructive, because other antibacterial drugs have failed to demonstrate adequate efficacy in NP/VAP, possibly as a consequence of such analyses being incomplete prior to clinical trials [10].

## Ceftazidime–avibactam: development overview

Ceftazidime–avibactam combines the established cephalosporin ceftazidime (which, among other indications, is approved as a monotherapy for HAP/VAP caused by susceptible bacteria), with avibactam, a novel, non-β-lactam inhibitor of Ambler class A, class C, and some class D β-lactamases [19]. The combination is active against a wide range of Gram-negative bacteria, including most carbapenemase-resistant Enterobacteriaceae (with the exception of those producing metallo-β-lactamases), and some multidrug-resistant *P. aeruginosa* strains [20–22].

The efficacy and safety of ceftazidime–avibactam, including in patients with infections caused by ceftazidime-resistant bacteria, have been demonstrated in a comprehensive adult clinical trial program, including two phase II [23, 24] and five phase III trials [25–29]. The phase II trials, and the first four phase III trials, enrolled adults with complicated intra-abdominal infection (cIAI) or complicated urinary tract infection (cUTI), and supported initial US (in 2015) and European (in 2016) approvals in these indications [30, 31]. Population PK modeling and probability of target attainment (PTA) analyses, using phase I and II clinical PK data and PK/PD targets derived from preclinical data (discussed below), were used to select a ceftazidime–avibactam dosage of 2000/500 mg by 2-h intravenous infusion q8h for patients with normal renal function across all of the phase III trials. The phase III trials all included sparse PK sampling schedules, and at various stages during the development program, additional patient PK and covariate data were incorporated into updated iterations of the ceftazidime and avibactam population PK models to evaluate the performance of the selected dose by assessing model predictions versus actual exposures. These analyses provided assurance that the selected regimen would provide adequate exposures for various clinically important patient subgroups, including patients with NP/VAP [31–33], and also supported selection of dosage adjustments for renal function [34, 35]. In conjunction with microbiological surveillance data and data from in vitro and animal models, the simulations also supported determination of ceftazidime–avibactam MIC susceptibility breakpoints [36].

The selected ceftazidime–avibactam dosage regimen for patients with NP/VAP was based on a PK/PD-guided approach, described here in detail, and led to European approval of ceftazidime–avibactam for adults with HAP/VAP before the completion of the final phase III trial (REPROVE), which evaluated ceftazidime–avibactam compared with meropenem in patients with NP, including VAP [29, 37]. Since there was no clinical experience with ceftazidime–avibactam in NP/VAP prior to REPROVE, the study also served to validate the dosage selection approach in a clinical setting [29]. A US label extension to include adults with HAP/VAP [38] was granted following the completion of the trial and analyses according to US Food and Drug Administration (FDA)–specified endpoints [39].

## Establishing PK/PD targets for NP and VAP

Population PK modeling and PTA analyses using PK/PD targets derived from preclinical data are commonly used to guide and support the selection of appropriate antibiotic dosage regimens for clinical use [40–43]. Achievement of 50% free drug time above the MIC (*f*T>MIC) is an established PD target for ceftazidime that is associated with up to 2 log_10_ killing of Enterobacteriaceae and *P. aeruginosa* in neutropenic mouse infection models [44–46], and with microbiological eradication in patients with NP caused by Gram-negative pathogens [47, 48]. This has led to the use of 50% *f*T>MIC as the key exposure target for evaluating ceftazidime PK/PD target attainment and in establishing MIC interpretive criteria (breakpoints) [36, 49, 50]. For avibactam, hollow-fiber and murine thigh and lung infection models with ceftazidime-resistant Enterobacteriaceae and *P. aeruginosa* were used to determine the avibactam PK/PD index in combination with ceftazidime [51, 52]. Based on these experiments, the avibactam PK/PD target in plasma associated with restoration of ceftazidime activity was defined as 50% free time above a critical avibactam threshold concentration of 1 mg/L (%*f*T>C_T_ 1 mg/L) during each dosage interval [33, 51, 52].

For dosage selection in NP, including VAP, it was necessary to study the lung penetration of ceftazidime and avibactam to establish whether these plasma-based PK/PD targets derived from murine infection models were appropriate surrogates for the achievement of adequate ceftazidime–avibactam exposures in human ELF. Moreover, it was important to assess the in vitro activity of ceftazidime–avibactam against clinical bacterial isolates from patients with pneumonia and to evaluate any impact of pulmonary surfactant, a primary component of ELF, or the presence of other antibiotics on in vitro activity.

### Bridging the gap between mice and men: confirming the suitability of plasma PK/PD targets derived from murine models for dosage selection in NP and VAP

Successful microbiological eradication requires adequate drug concentrations at the site of infection. Therefore, the extent of penetration of ceftazidime and avibactam into ELF was an important consideration for appropriate dosing in the treatment of NP [10]; however, there are several limitations to this approach (see Supplemental Materials), and in particular it may underestimate the extent of pulmonary penetration of β-lactams [53].

Murine infection models are commonly used to identify PK/PD targets, as it is difficult to determine the PK/PD index from clinical trials, which often have too few clinical failures to conduct exposure–response analyses [54]. However, bridging PK data from mice to humans without considering human ELF data may result in incorrect calculations for the drug exposure expected at the infection site [10].

**Fig. 1:**
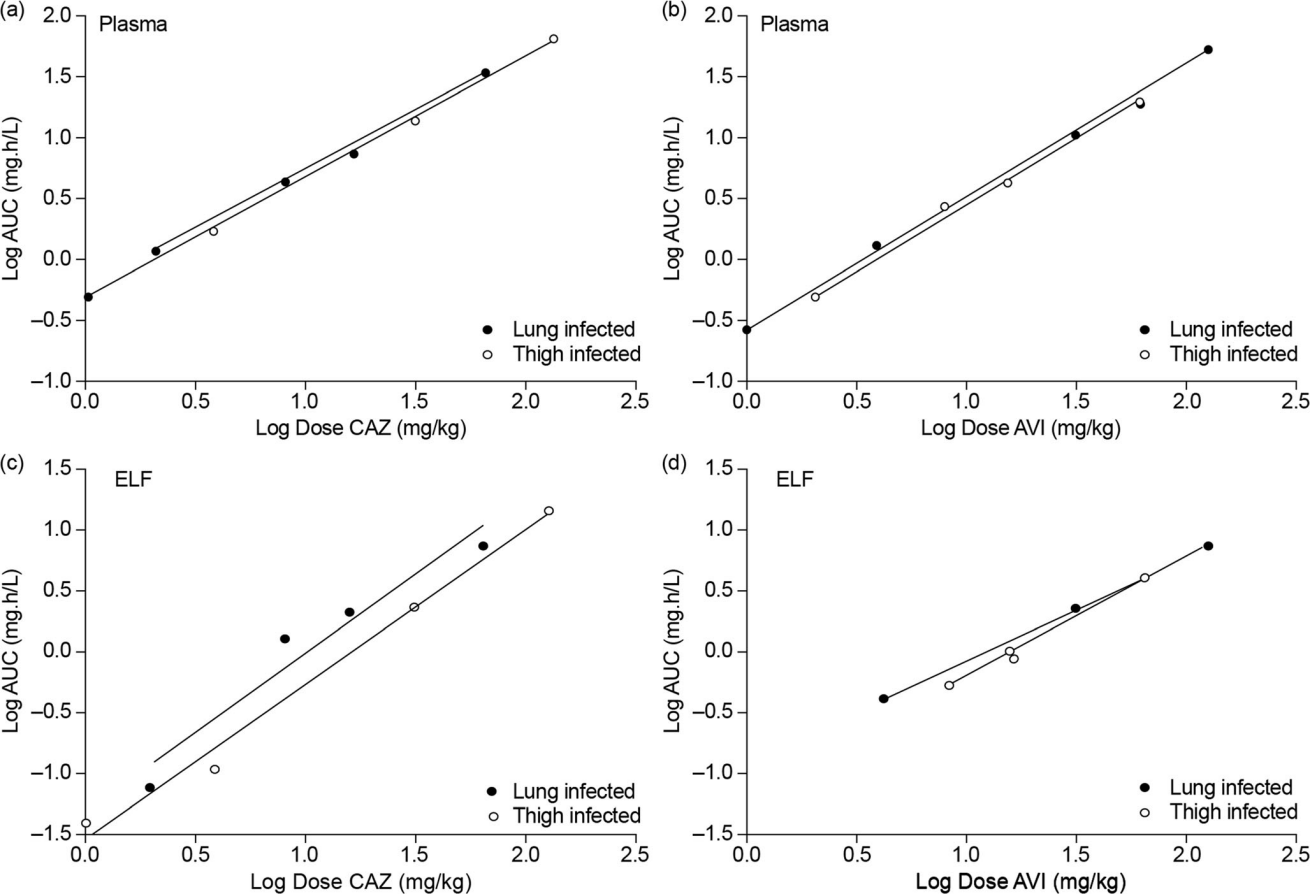
Murine model: dose proportionality of ceftazidime (parts **a**, **c**) and avibactam (parts **b**, **d**) in plasma (parts **a**, **b**) and ELF (parts **c**, **d**) in neutropenic mice infected in the thigh or lung by *Pseudomonas aeruginosa. AUC* area under the concentration–time curve, *AVI* avibactam, *CAZ* ceftazidime, *ELF* epithelial lining fluid. Dose, a single dose administered subcutaneously. Figures from Berkhout et al. [55]

**Fig. 2:**
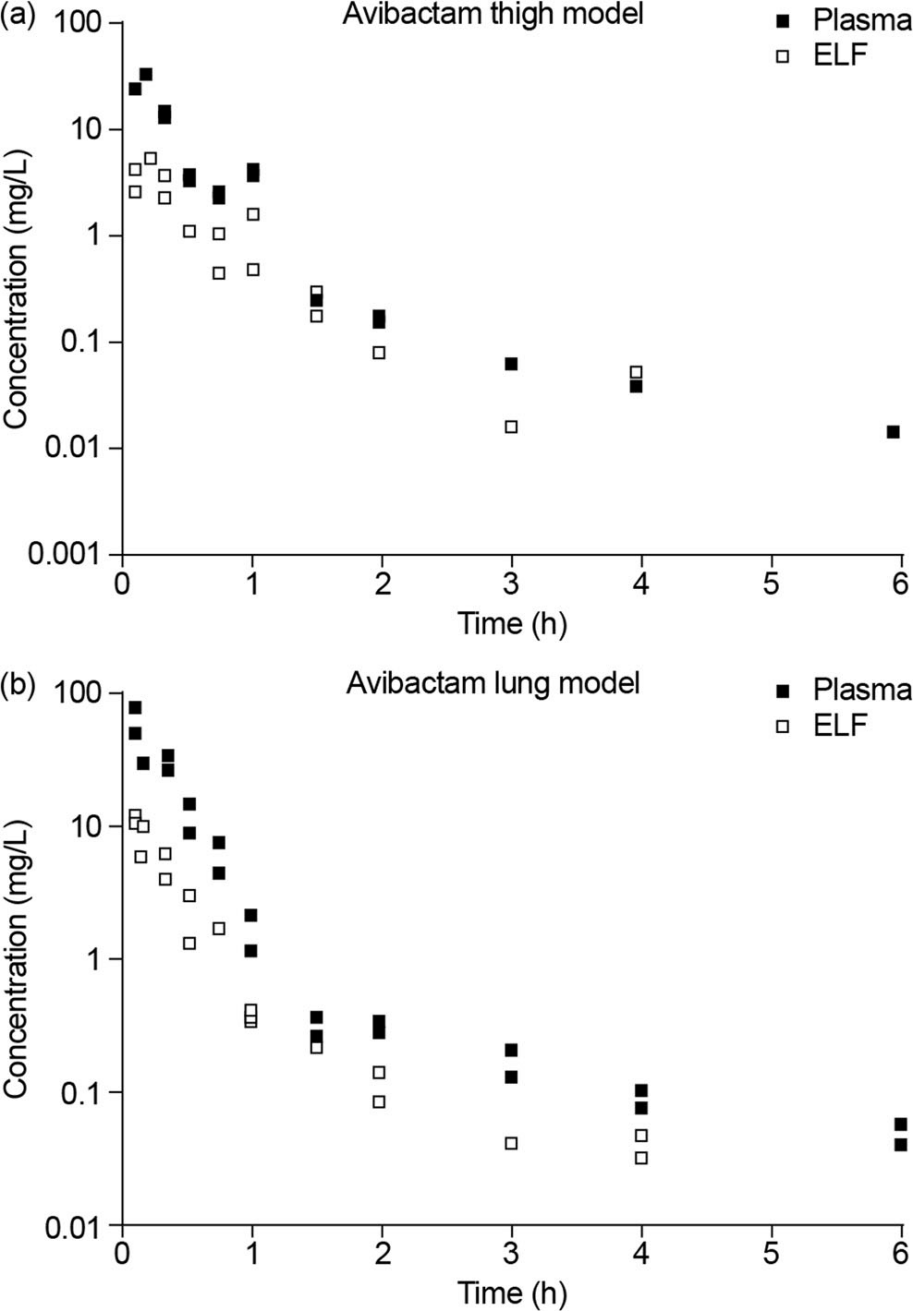
Murine model: example of PK profiles for two different single doses of avibactam (co-dosed with ceftazidime) in plasma and ELF of neutropenic mice with **a** thigh infection (32 mg/kg dose) or **b** lung infection (64 mg/kg dose). *ELF* epithelial lining fluid, *PK* pharmacokinetic. Each dose group consists of two mice. Figures from Berkhout et al. [55]

**Fig. 3:**
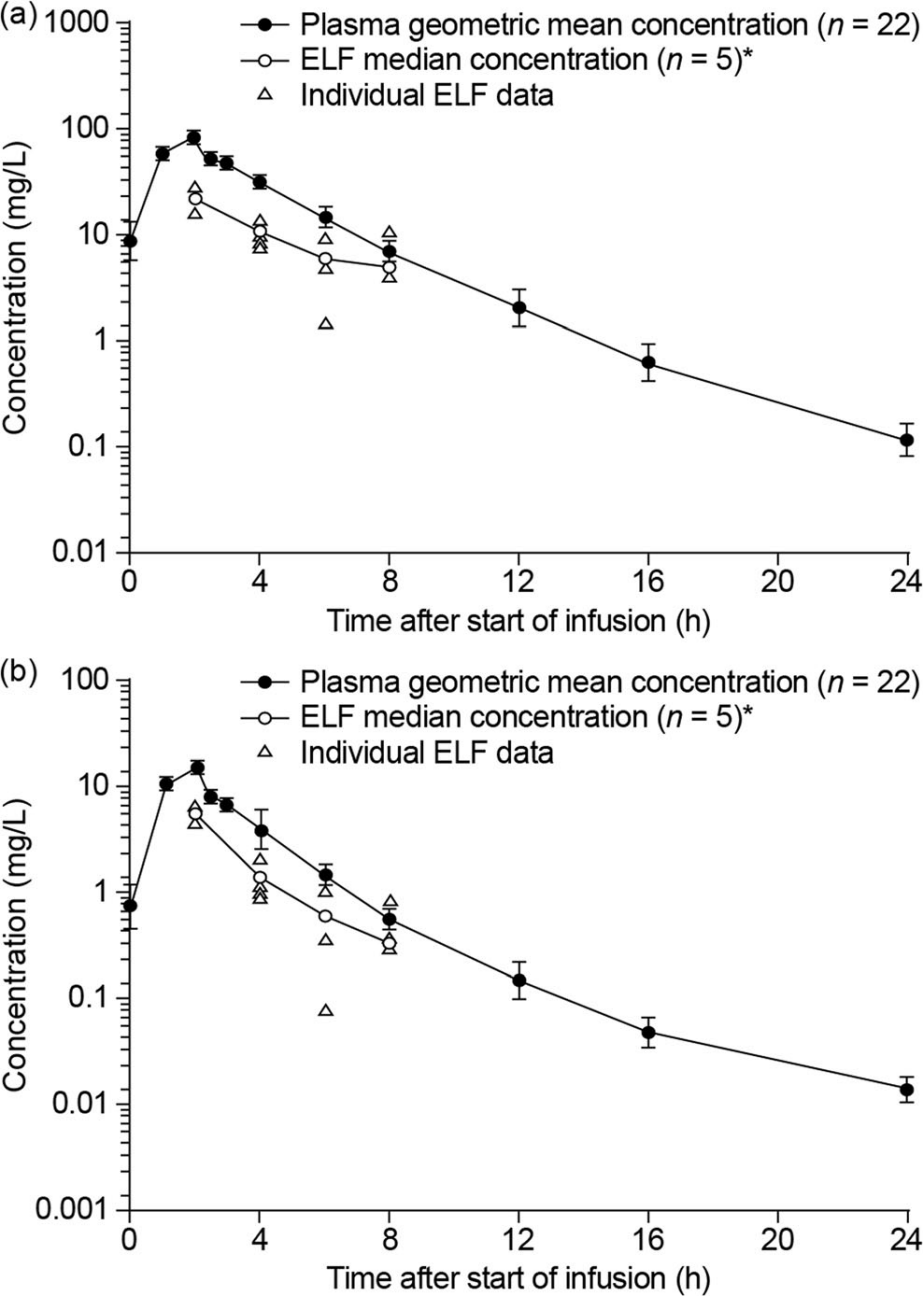
Human volunteer study: geometric mean (± SD) plasma and median and individual ELF concentration–time profiles for **a** ceftazidime 2000 mg and **b** avibactam 500 mg (semi-logarithmic scale). *ELF* epithelial lining fluid, *SD* standard deviation. *n = 6 for ELF median concentrations at 2 h and 4 h in the 2000/500 mg ceftazidime–avibactam group. Figure from Nicolau et al. [56]

Plasma and ELF PK data for ceftazidime–avibactam from a phase I trial that sampled ELF in healthy volunteers were compared with plasma and ELF PK data obtained from murine neutropenic thigh and lung infection models [55, 56]. In the human phase I study, ELF/plasma AUC ratios were calculated using total (free + bound) plasma concentrations of ceftazidime and avibactam (i.e., they were not corrected for differences in protein binding in plasma and ELF) [56], resulting in slightly lower estimated human ELF/plasma exposure ratios than if calculated using unbound fractions. In contrast, the murine ELF/plasma AUC ratios accounted for protein binding of ceftazidime and avibactam, and were calculated using free concentrations (protein binding is approximately 10% for both ceftazidime and avibactam in plasma, and negligible in ELF) [53, 55]. Both ceftazidime and avibactam penetrated similarly into mouse (Figs. 1 and 2) and human ELF (Fig. 3) in a dose-proportional manner [55, 56], and concentration-time profiles in ELF were a similar shape to those in plasma (Figs. 1 and 3). The mouse ELF:plasma AUC ratios for ceftazidime and avibactam (20–24%) were slightly lower than the corresponding human values (31–35%) [55, 56], suggesting that the mouse data provided a conservative indication of ELF penetration achieved in humans. Thus, as lower penetration in mouse still resulted in effective bacterial killing, although ELF concentrations in humans were a proportion of those in plasma, these were considered sufficient to achieve adequate free lung exposures.

These findings, in addition to studies showing a lack of interaction between pulmonary surfactant and ceftazidime or avibactam (see below), and of interactions with other antibiotics (see Supplemental Materials), indicated that ceftazidime and avibactam PK in plasma are appropriate surrogates for the PK of these drugs in ELF and confirmed the suitability of the mouse infection model to derive plasma-based PK/PD targets for estimation of PTA appropriate to support dosage selection in NP.

### In vitro activity of ceftazidime–avibactam against bacteria isolated from patients with non-ventilated or ventilated pneumonia

**Fig. 4:**
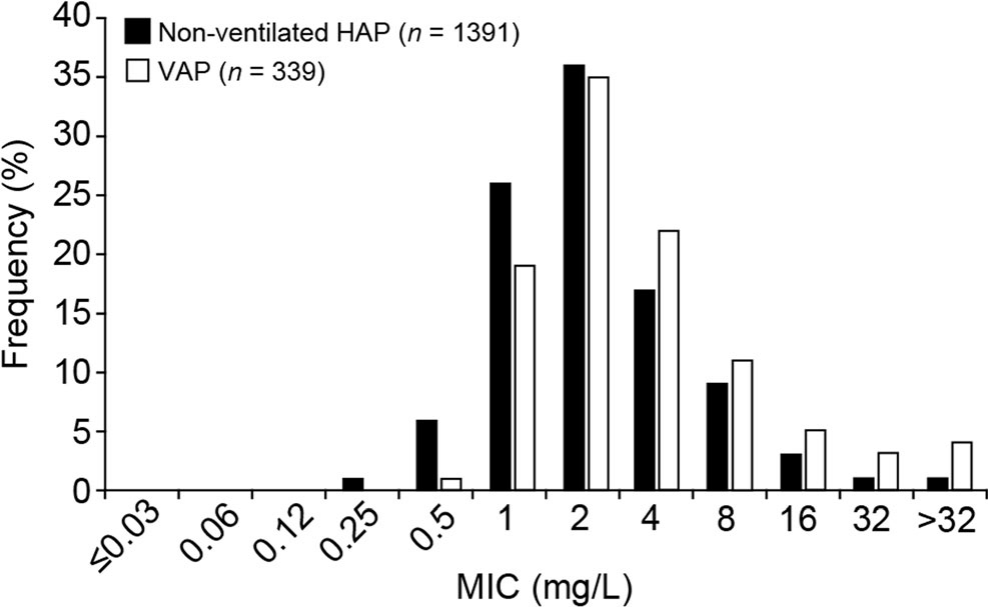
Ceftazidime–avibactam MIC distribution of *Pseudomonas aeruginosa* isolates from non-ventilated and ventilated hospitalized patients with pneumonia. *HAP* hospital-acquired pneumonia, *MIC* minimum inhibitory concentration, *VAP* ventilator-associated pneumonia. Data sourced from study conducted by Flamm et al. [57]

To account for potential differences in susceptibility among bacteria causing NP and VAP compared with other infection types, the in vitro activity of ceftazidime–avibactam was evaluated against bacterial isolates from patients with pneumonia. In an international surveillance study, the ceftazidime–avibactam MIC was ≤ 8 mg/L in 92–96% of *P. aeruginosa* isolates from non-ventilated hospitalized patients with pneumonia, and in 79–95% of those obtained from ventilated patients (Fig. 4) [57]. Ceftazidime–avibactam MIC_90_ values for Enterobacteriaceae ranged from 0.25 to 0.5 mg/L [57]. This was concordant with analyses of isolates from patients with other infection types, which have reported ceftazidime–avibactam MIC_90_ values of ≤ 8 mg/L for *P. aeruginosa* and Enterobacteriaceae [22, 58–62]. A target-free plasma ceftazidime exposure (*f*T>MIC) based on high target attainment against bacteria that test with MIC of ceftazidime–avibactam of 8 mg/L was therefore considered appropriate for use in dosage selection for NP and VAP.

### In vitro interaction with pulmonary surfactant

Pulmonary surfactant, a complex lipid and protein mixture that is present in the ELF, binds to some antimicrobials, causing their activities to decrease [16]. Dallow and colleagues [63] conducted an in vitro study to evaluate the effect of pulmonary surfactant on the activity of ceftazidime–avibactam and to determine whether there was any antagonistic in vitro interaction with other antimicrobial classes that are commonly used in the treatment of NP. No significant increases in ceftazidime–avibactam MIC were observed at pulmonary surfactant concentrations up to 10% for β-lactamase-producing Gram-negative bacteria, indicating that pulmonary surfactant does not adversely affect the in vitro activity of ceftazidime–avibactam [63].

## Population PK modeling and PTA analyses to guide dosage selection for NP and VAP

Population PK models were developed for ceftazidime and avibactam using patient PK data from clinical trials and updated as additional data were generated throughout the clinical development program, with key iterations including two early phase I–II models [64, 65], two interim phase I–III models [66, 67], and a final model [32], with each new iteration incorporating all previously available data; the model iterations have been reviewed in detail by Das et al. (2019) [68], and details of the final model, including NONMEM codes, as well as descriptions of the model construction, selection of covariates, and model evaluation, have been reported by Li et al. (2019) [32]. Ceftazidime and avibactam plasma concentration–time data were analyzed using nonlinear mixed-effects modeling, which described the PK of both ceftazidime and avibactam as a two-compartment disposition model with first-order elimination from a central compartment following intravenous infusion, parameterized by clearance (CL), volume of the central compartment (*V*_c_), intercompartmental clearance (*Q*), and volume of the peripheral compartment (*V*_p_) [32]. The final model parameter estimates are shown in the Supplementary Materials. Prediction-corrected visual predictive checks confirmed that the various model iterations reflected the observed data [32, 68]. These models were used in Monte Carlo simulations to support and validate dosage selection, with simulation methods summarized in the Supplementary Materials. Based on the in vitro and in vivo studies described above, free plasma PK/PD targets of 50% *f*T > 8 mg/L for ceftazidime (8 mg/L was selected as it was the ceftazidime–avibactam MIC_90_ for the target species, *P. aeruginosa*, and higher than the MIC_90_ for species of the Enterobacteriaceae, as described above) and 50% *f*T > C_T_ 1 mg/L for avibactam (see Nichols et al. (2018) [33] for a review of PK/PD target selection), to be attained simultaneously in each simulated patient, were used in PTA analyses to guide dosage selection based on achievement of > 90% joint PTA [31, 32, 68].

The overall phase III dosage selection and optimization process for ceftazidime–avibactam has been reviewed by Das et al. [68]. In brief, ceftazidime 2000 mg q8h was selected as the “starting point,” as this is the recommended monotherapy dose for severe infections [69], co-administered with avibactam 500 mg q8h in a 4:1 fixed-dose ratio. At the time of dosage selection for REPROVE, PK data for ceftazidime–avibactam were available from five phase I studies in healthy volunteers and a phase II study in patients with cIAI treated with ceftazidime–avibactam 2000/500 mg 30-min intravenous infusions q8h. Ceftazidime and avibactam population PK models were developed using data from these studies and used to guide dosage selection for REPROVE, because no clinical trial PK data were available at the time for ceftazidime–avibactam in patients with NP/VAP. For both ceftazidime and avibactam, the final models were two-compartmental, with body-surface-area-normalized creatinine clearance (CL_CR_), age, body weight, and study population (healthy subjects vs patients) identified as covariates [64, 70]. The use of population PK models based on patients with cIAI was considered appropriate for dosage selection in NP, as the PK profile of ceftazidime alone was previously shown to be comparable across cIAI and NP patient populations [71, 72]. We assumed that the same would be true for avibactam, given the generally similar PK profiles of the two compounds [73].

ARC occurs commonly in patients who are critically ill, such as those with VAP, resulting in faster elimination of renally cleared drugs [10, 15]. As both ceftazidime and avibactam are almost entirely cleared by the kidneys, and CL_CR_ was identified as a significant covariate affecting the exposures of both drugs in the population PK models, the effect of high CL_CR_ was an important consideration in dosage selection for NP/VAP [10]. Following completion of the ceftazidime–avibactam phase III cIAI and cUTI trials, updated population PK models were developed which included data from 101 phase III patients with estimated CL_CR_ 150–180 mL/min and 76 with CL_CR_ 180–395 mL/min [31]. These updated models were used to explore specific considerations for the CL_CR_ distribution in simulations of patients with NP/VAP [70]. We assumed that the relationships between ceftazidime and avibactam exposures and high CL_CR_ were already well characterized in the models based on patients with cIAI. This assumption was supported by data from a phase I study which explored the impact of ARC on avibactam exposure when dosed in combination with ceftaroline fosamil in patients with confirmed ARC (measured CL_CR_ ≥ 140 mL/min) and sepsis [31]. An increase in avibactam CL in patients with ARC and sepsis was seen compared with healthy subjects (noncompartmental analysis), resulting in an average 28.4% lower AUC_0–*t*_, which was comparable to the avibactam exposure seen in phase III patients with cIAI with estimated CL_CR_ > 150 mL/min [31]. There was a similar impact of CL_CR_ > 150 mL/min on the exposure of ceftazidime. The similar magnitudes of differences in exposures (compared to healthy subjects with normal renal function) between patients with cIAI with high estimated CL_CR_ (> 150 mL/min) and patients with ARC and sepsis suggested that it was plausible to use estimations of high CL_CR_ from the phase III data to model the impact of ARC on PTA.

**Fig. 5:**
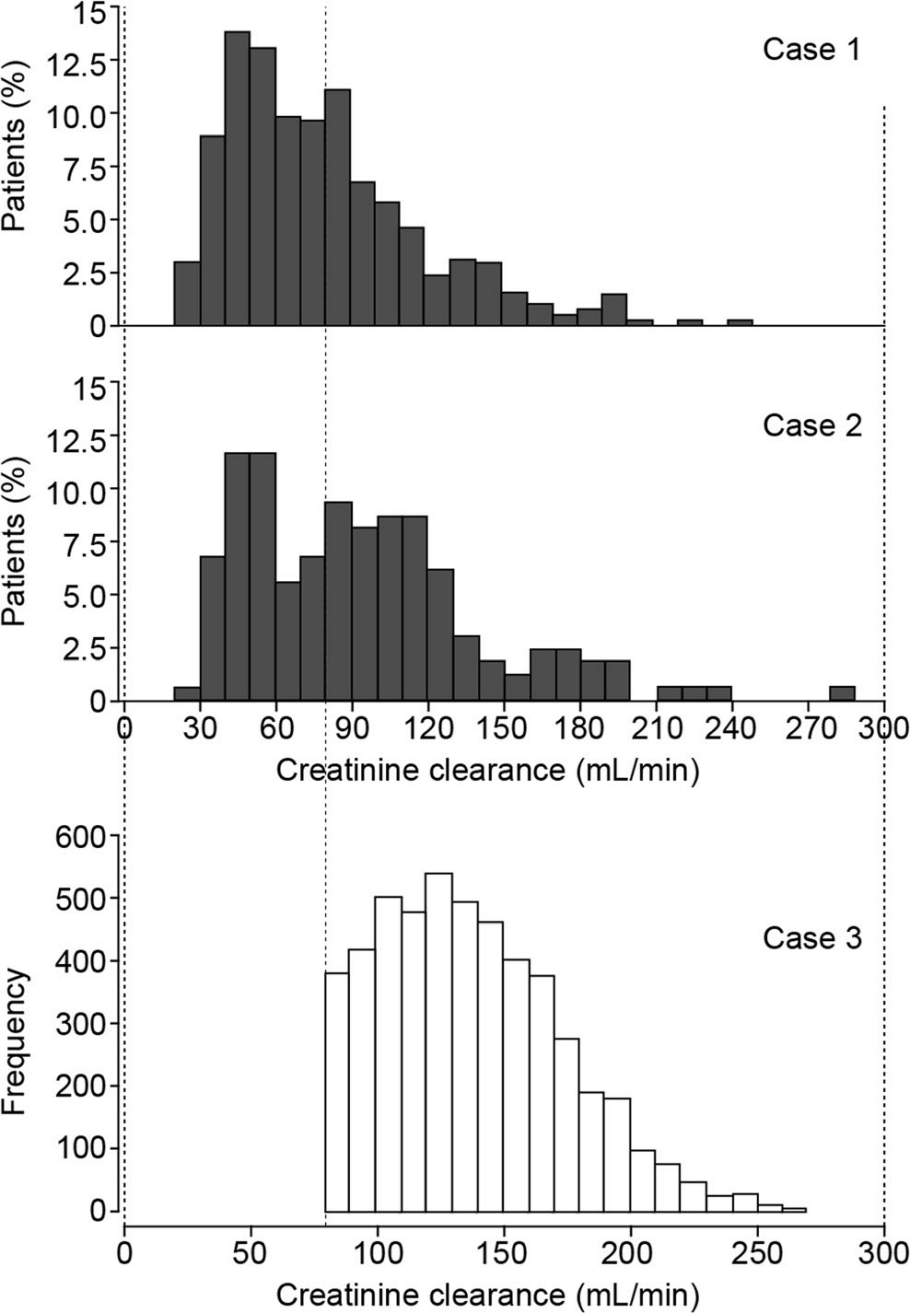
Patient CL_CR_ distributions used in the PTA simulations for dosage regimen selection in NP including VAP. *CL*_*CR*_ creatinine clearance, *NP* nosocomial pneumonia, *PTA* probability of target attainment, *VAP* ventilator-associated pneumonia. Case 1: CL_CR_ distribution from 415 non-ventilated patients with NP [10]. Case 2: CL_CR_ distribution from 164 patients with VAP [10]. Case 3: CL_CR_ distribution from the phase III CANVAS 1 and 2 studies of ceftaroline fosamil in complicated skin and soft-tissue infection. This distribution was used as these trials contained many patients with infection and high CL_CR_ (truncated at CL_CR_ > 80 mL/min). Dashed lines show the alignment of the distribution. Data from Li et al. (2015) [70]. Simulations were conducted for 3000 patients each in case 1 and case 2 (of which 2000 simulated subjects had CL_CR_ > 80 mL/min and 1000 had CL_CR_ 50–80 mL/min), and 1000 patients in case 3, receiving ceftazidime–avibactam 2000/500 mg q8h 2-h intravenous infusions. Case 1 and 2 panels adapted from Ambrose et al. (2010) [10]

To assess the impact of higher renal clearance in patients with NP, additional simulations based on the updated models were conducted to evaluate whether the proportion of patients with high CL_CR_ significantly affected PTA [70]. During typical PTA simulations, the CL_CR_ distribution is re-sampled using the population model patient dataset. In the original simulations for NP dose selection, the population PK models were developed using data from patients with cIAI, and did not include patients with NP. Therefore, the impact of high CL_CR_ on PTA was analyzed in three scenarios (Fig. 5). First, the CL_CR_ distribution from patients with cIAI was replaced with literature-reported values from patients with NP, using CL_CR_ distributions from non-ventilated (case 1) and ventilated (case 2) patients [10]. A third approach (case 3), simulated a “high CL_CR_ distribution” by removing low clearances from a natural patient distribution (i.e., of the CL_CR_ range seen in a clinical trial, the distribution of values > 80 mL/min was taken and re-sampled). In this instance, the CL_CR_ distribution was derived from two phase III studies of ceftaroline fosamil in complicated skin and soft-tissue infection [74]. These two studies included multiple subjects with infection and high CL_CR_, and were therefore considered informative for simulations of patients with NP (Fig. 5). In this distribution, at least 25% of patients had CL_CR_ > 160 mL/min and could be considered to have ARC. Other covariates (age, weight, height, and gender) were simulated from their distribution functions obtained from the phase II trial in patients with cIAI [24, 70].

For each CL_CR_ distribution case defined above, joint PTA by MIC curves were compared with ceftazidime–avibactam MIC distributions for *P. aeruginosa* isolates from patients with NP and VAP (Fig. 4) [57]. Joint PTA > 90% was predicted for ceftazidime–avibactam 2000/500 mg 2-h intravenous infusions q8h for all three CL_CR_ distribution cases (NP, VAP, and high CL_CR_) for ceftazidime–avibactam MICs ≤ 8 mg/L (Table 1) [70]. These simulations thus supported the selected ceftazidime–avibactam dosage regimen for patients with NP, including VAP, and CL_CR_ ≥ 50 mL/min. Dosage adjustments are required for patients with CL_CR_ < 50 mL/min, as described elsewhere [32].

**Table 1:** Predicted joint PTA (%) by ceftazidime–avibactam MIC for patients receiving ceftazidime–avibactam 2000/500 mg q8h as a 2-h intravenous infusion for three different cases of CL_CR_ distributions (dose selection population PK model)

Ceftazidime–avibactam MIC (mg/L)	Non-ventilatedNP patients^a^ (case 1)	VentilatedNP patients^a^ (case 2)	Patients with high CL_CR_^b^ (case 3)
0.125	98.2	98.2	97.9
0.25	98.2	98.2	97.9
0.5	98.2	98.2	97.9
1	98.2	98.2	97.9
2	98.2	98.2	97.9
4	98.0	98.2	97.7
8	95.7	94.8	92.5
16	65.6	63.8	53.8
32	12.3	12.1	6.3

*CL*_*CR*_ creatinine clearance, *MIC* minimum inhibitory concentration, *NP* nosocomial pneumonia, *PK* pharmacokinetic, *PTA* probability of target attainment, *q8h* every 8 h

^a^For case 1 (non-ventilated NP) and case 2 (ventilated NP) simulations, 3000 patients were simulated for each case, of which 2000 were simulated from the distribution region CL_CR_ > 80 mL/min, and 1000 from the region CL_CR_ 50–80 mL/min; covariate distributions for CL_CR_ were obtained from the literature [10]

^b^For case 3, 1000 patients were simulated; the covariate distribution for CL_CR_ was obtained from two phase III studies of ceftaroline fosamil in patients with complicated skin and soft-tissue infection with high CL_CR_, excluding values < 80 mL/min (case 3). See Fig. 5 for details

Data from Li et al. (2015) [70]. Simulations were conducted for 3000 patients each in case 1 and case 2 (of which 2000 simulated subjects had CL_CR_ > 80 mL/min and 1000 had CL_CR_ 50–80 mL/min), and 1000 patients in case 3, receiving ceftazidime–avibactam 2000/500 mg q8h 2-h intravenous infusions

## Approval of ceftazidime–avibactam for the treatment of NP and VAP in Europe prior to the completion of REPROVE

**Fig. 6:**
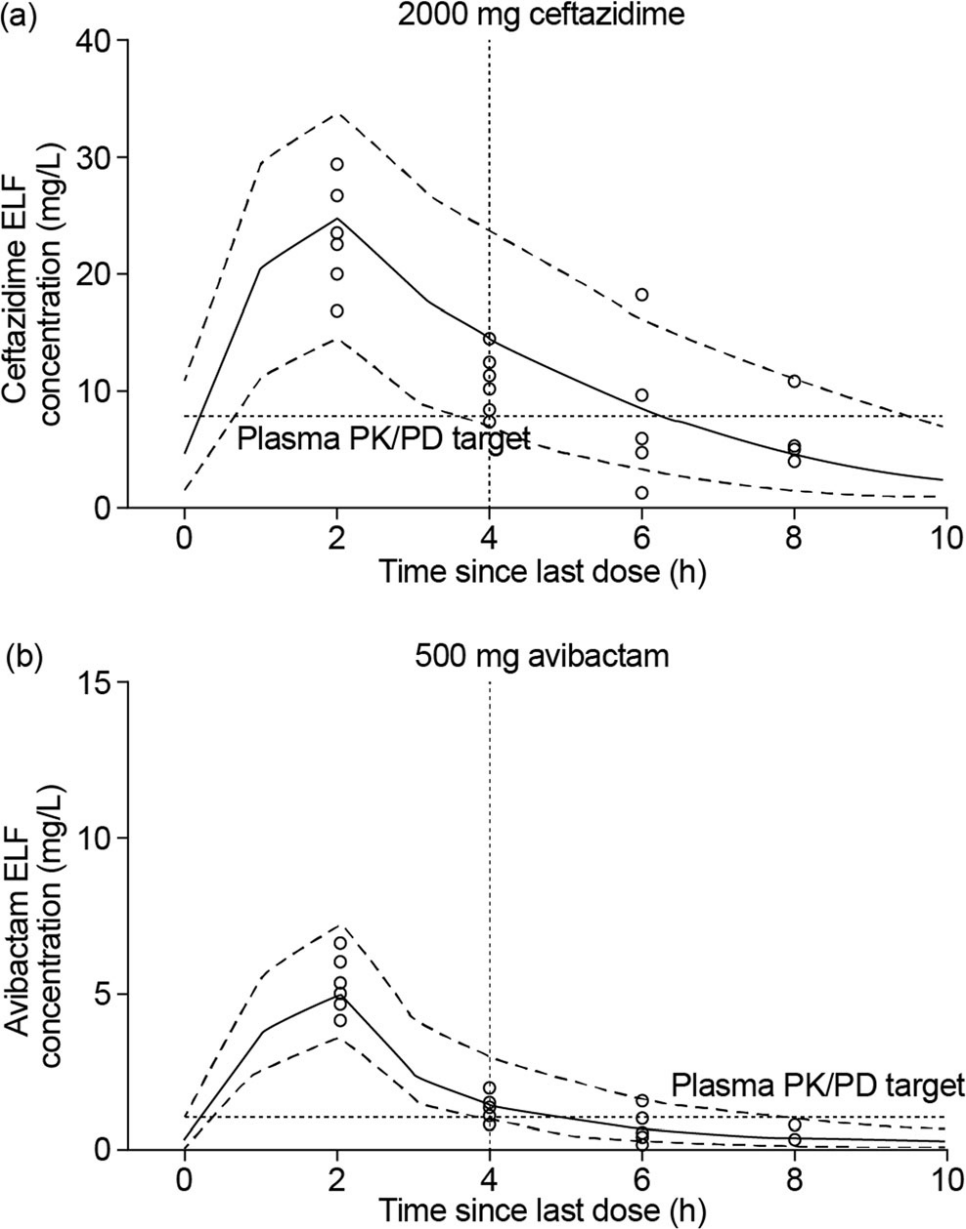
Simulated total ELF concentration–time profiles in 1000 subjects receiving ceftazidime–avibactam 2000/500 mg q8h for **a** ceftazidime and **b** avibactam, superimposed with observed ELF concentration data. *ELF* epithelial lining fluid, *MIC* minimum inhibitory concentration, *PK/PD* pharmacokinetic/pharmacodynamic. The solid line represents the median percentile of 1000 simulated individuals, and the dashed lines are 5th and 95th percentiles. The circles represent the observed data points. The horizontal hashed line represents the plasma PK/PD target for ceftazidime (50% *f*T>MIC of 8 mg/L) and avibactam (50% *f*T > 1 mg/L), and the vertical hashed line represents the midpoint of the 8-h dosing interval. Figure from Dimelow et al. (2018) [75]

Ceftazidime–avibactam was approved in Europe for the treatment of HAP, including VAP, prior to the completion of REPROVE [31, 37]. This decision was supported by the PK analyses described above and the following additional data. At the time of the European approval in HAP/VAP, results from the RECLAIM, REPRISE, RECAPTURE, and RECLAIM 3 phase III clinical trials had confirmed the efficacy in patients with cIAI and cUTI of the proposed NP dosage regimen [25–28]. Blinded interim plasma concentration data were also available from 308 patients (109 with VAP) from REPROVE, which demonstrated similar ceftazidime and avibactam plasma exposures in patients with NP or VAP compared with those for patients with cIAI [31]. Updated ceftazidime and avibactam population models, including data from the phase III clinical trials in cIAI and cUTI, were used together with blinded demographic data from patients with NP and VAP in REPROVE to simulate joint PTA for patients with NP, including VAP, and confirmed high joint PTA (> 98%) in these patients at ceftazidime–avibactam MICs up to 8 mg/L [31]. Ceftazidime and avibactam plasma–ELF relationships were also further characterized by developing population PK models for ceftazidime and avibactam in plasma and ELF using data from the phase I ELF study [56, 75]. ELF penetration of ceftazidime and avibactam from plasma occurred rapidly and was nonlinear, with penetration of both drugs greater than previously calculated using noncompartmental AUC methods [75]. Simulation of ELF concentration–time profiles demonstrated that most subjects achieved ceftazidime and avibactam ELF exposures exceeding their respective plasma PK/PD targets by the midpoint of the dosing interval (Fig. 6). This analysis confirmed that substantial ELF penetration is achieved in healthy subjects, and that the exposures in the lungs of both drugs administered at the proposed dosage regimen were predicted to exceed levels required for efficacy.

## Validation of the ceftazidime–avibactam dosage regimen for patients with NP, including VAP

In REPROVE, 879 adults with NP, including VAP, were randomized 1:1 to receive ceftazidime–avibactam 2000/500 mg 2-h intravenous infusion q8h or meropenem 1000 mg 30-min infusion q8h, with dosage regimens of both drugs adjusted for impaired renal function [29, 68]. Ceftazidime–avibactam was non-inferior to meropenem with respect to clinical cure at test of cure (primary endpoint) [29] and for the US FDA–specified endpoint of all-cause mortality at day 28 [39]. Adverse events were as expected for the patient population and consistent with the established safety profile of ceftazidime–avibactam [29, 39]. Of note, ceftazidime–avibactam dosage adjustments for CL_CR_ < 50 mL/min were modified by protocol amendment during the study to reflect approved European and US labeling [68]. Patients in REPROVE receiving the original dosage adjustments were excluded from the primary efficacy analyses but not from the US analyses; however, in both cases, the non-inferiority criteria were met [29, 39]. Thus, the selected ceftazidime–avibactam dosage regimens (including recommended adjustments for impaired renal function) were associated with clinical efficacy in patients with NP, including VAP, validating the dosage selection approach.

As noted above, patients with NP, and particularly VAP, are often critically ill, which can affect the PK of many drugs, including some antibiotics [13]. Therefore, updated ceftazidime and avibactam population PK models, based on patient PK data from all five adult ceftazidime–avibactam phase III trials (including REPROVE), were used to confirm that appropriate exposures and high target attainment were maintained in patients with NP and VAP, and in subgroups of patients with characteristics of critical illness [32]. The final models included ceftazidime and avibactam PK data from 1975 and 2249 individuals, respectively, including 412 patients with NP, of whom 138 had VAP. A high proportion of patient PK data was included in these models, including patients with a wide range of renal function, and substantial numbers of patients with more severe infection (438 patients with Acute Physiology and Chronic Health Evaluation [APACHE] II score > 10, and 773 patients with systemic inflammatory response syndrome [SIRS] at baseline). Consistent with the earlier population PK models, CL_CR_ was the key covariate that affected CL of both drugs. The CL of avibactam and ceftazidime was close to proportional to CL_CR_ at CL_CR_ values < 80 mL/min and < 100 mL/min, respectively; at higher CL_CR_ values, CL increased modestly (shallow slope) as CL_CR_ increased.

The final models were used to predict steady-state exposures and joint PK/PD target attainment in actual phase III patients, and conduct joint PTA simulations for multiple patient subgroups including indication (cIAI, cUTI, or NP), baseline APACHE II score, presence of SIRS, renal function, age, sex, and race. Covariate values for simulations in the different indications were obtained by sampling with replacement from the corresponding set of phase III study patients for each indication (bootstrapped from the respective trial populations). Patients with NP were stratified into VAP and non-VAP patient subgroups for the covariate analysis. To further assess the effect of ventilation, an additional subgroup was defined for “patients with a ventilator in the hospital room,” which included patients with HAP or VAP who were ventilated on the day of PK sampling (denoted as NPv).

Predicted plasma ceftazidime and avibactam exposures in phase III patients with NP (including non-VAP, VAP, and NPv subgroups) were broadly comparable with (but slightly higher than) exposures in patients with cIAI and cUTI. Joint PK/PD target attainment exceeded 97% in all NP subgroups, as well as in patients with markers of severe disease, including APACHE II > 10, SIRS, or bacteremia [32]. In phase III patients with high (151–180 mL/min) or very high (181–610 mL/min) CR_CL_, predicted joint PK/PD target attainment rates were 98.4% and 95.7%, respectively, reflecting the relatively small increases in ceftazidime and avibactam CL at higher CL_CR_ values [32]. These findings, consistent with the clinical outcomes, suggest that the selected dosage regimen provided appropriate exposures and high joint target attainment in patients with NP, including VAP, regardless of infection severity or ARC.

In simulations based on the final models and covariate distributions, exposures were highest in patients with NP (compared to those with cIAI or cUTI), and PTA > 95% at a ceftazidime–avibactam MIC of 8 mg/L was predicted for patients with NP/VAP/NPv, including those with high CL_CR_ (Table 2). Appropriate exposures and > 96% joint PTA were also predicted across renal function categories in patients with NP receiving the recommended ceftazidime–avibactam dosage adjustments for renal impairment [32].

**Table 2:** Simulated geometric mean (CV%) steady-state exposures and joint PTA for ceftazidime and avibactam summarized by indication and by NP subgroup for patients with normal renal function (dose validation population PK model)

	Ceftazidime		Avibactam		Joint PTA, %
	*C*_max,ss_ (mg/L)	AUC_ss,0–24_ (mg·h/L)	*C*_max,ss_ (mg/L)	AUC_ss,0–24_ (mg·h/L)	
cIAI	61.1 (44)	683 (45)	11.5 (83)	121 (72)	94.9
cUTI	73.0 (47)	880 (49)	11.2 (87)	126 (82)	95.2
NP	65.4 (53)	805 (55)	12.8 (94)	147 (89)	98.3
NPv	56.8 (51)	723 (56)	11.2 (82)	131 (75)	97.2
VAP	55.1 (59)	719 (64)	10.7 (85)	129 (79)	96.1
Non-VAP	75.7 (43)	894 (48)	14.7 (92)	164 (93)	100

*AUC*_*ss,0–24*_ area under the concentration–time curve at steady state, *C*_*max,ss*_ maximum plasma concentration at steady state, *cIAI* complicated intra-abdominal infection, *CL*_*CR*_ creatinine clearance, *cUTI* complicated urinary tract infection, *CV* coefficient of variation, *NP* nosocomial pneumonia, *NPv* patients with a ventilator in the hospital room, which included patients with hospital-acquired pneumonia (HAP) or ventilator-associated pneumonia (VAP) who were ventilated on the day of PK sampling; *PK* pharmacokinetic, *PTA* probability of target attainment, *q8h* every 8 h

Data from Li et al. (2019) [32]. Simulations were conducted for 5000 patients with normal renal function (CL_CR_ > 80 mL/min) in each indication, receiving ceftazidime–avibactam 2000/500 mg q8h as a 2-h intravenous infusion

## Perspectives on selecting antibiotic dosage regimens for the treatment of NP and VAP

In selecting an appropriate dosage of ceftazidime–avibactam for use in patients with NP and VAP, we followed Ambrose and colleagues’ advice to “look before you leap” [10]. Specifically, we considered ceftazidime–avibactam MIC distributions against bacteria isolated from patients with pneumonia, drug penetration to the infection site, potential antagonism by lung surfactant (and antagonistic interaction with other antimicrobials [63]), the suitability of using murine-derived plasma PK/PD targets for human dosage selection, and the phenomenon of ARC that may occur in critically ill patients. These analyses had an important role in supporting the approval of ceftazidime–avibactam in Europe for the treatment of patients with HAP, including VAP [37], prior to the availability of clinical data in this indication. Results from the REPROVE trial subsequently confirmed the efficacy and safety of the approved ceftazidime–avibactam dosage regimen in patients with NP, including VAP [29, 39]. In 2018, ceftazidime–avibactam was approved in the USA for the treatment of HAP and VAP based on data from REPROVE [38].

Notwithstanding the successful outcome of dosage selection for ceftazidime–avibactam reported here, there were some limitations to the overall modeling approach, and in the data available for the population PK analyses. For example, we did not use a joint population PK model, which would have allowed for parametric simulations with consideration of PK parameter correlations. Moreover, the lack of ELF data from patients with lung infections required extrapolation from murine data and modeling of ELF penetration based on plasma concentrations. While recognizing the limitations of ELF measurements, we believe that the ceftazidime and avibactam ELF population PK models and exposure calculations can be considered conservative, and the positive outcome of the REPROVE trial provides further validation of the view that adequate lung tissue penetration is achieved in patients. Initial dosage selection was based on PK data from subjects with a limited range of CL_CR_, with updated models based on phase III cIAI data subsequently used for evaluating exposures and PTA in patients with NP/VAP, a population with highly variable renal function, including ARC. In addition, there are limitations in the use of the Cockcroft–Gault formula for estimation of renal function in subjects with high CL_CR_. Similarly, there are known effects of mechanical ventilation on the PK of multiple drugs, including antibiotics. Our analysis identifying patients in REPROVE as NPv, VAP, or non-VAP was based on the presence of a ventilator in the hospital room (using available study data), which limited exploration of the impact of mechanical ventilation. However, it should again be emphasized that the results from REPROVE support the selected ceftazidime–avibactam dosage as efficacious in patients with NP and VAP. Finally, owing to restrictions in the available clinical data, the population PK models were unable to provide specific exposure and PTA predictions for some patient groups of clinical interest, such as those with neutropenia. However, ceftazidime monotherapy is approved for the treatment of febrile neutropenia, and since the action of avibactam is independent of neutrophils, it is reasonable to expect that the ceftazidime–avibactam dosages, PK/PD targets, and exposures described here would also apply in patients with neutropenia with infections caused by β-lactamase-mediated ceftazidime-resistant (but ceftazidime–avibactam susceptible) bacteria.

## Conclusions

Selection of inappropriate dosage regimens is likely to have contributed to the failure of some antibiotic development programs in the NP and VAP indications [10]. As Ambrose and colleagues have suggested, this can potentially be avoided by considering the confounders and determinants of response in this patient population and conducting appropriate experiments to inform dosage regimen decisions [10]. Based on the work described here, ceftazidime–avibactam 2000/500 mg 2-h intravenous infusion q8h (adjusted for renal function) was the dosage regimen selected for patients with NP, including VAP, and which was evaluated in the phase III REPROVE trial. The results from REPROVE confirmed that the selected ceftazidime–avibactam dosage regimen was associated with efficacy in patients with NP, including VAP, validating the approach described here for dosage selection in REPROVE. The present work serves as a clinically successful example of designing dosing for clinical trialing of a new antibacterial agent in the “perfect storm” of NP, including VAP.

## Electronic supplementary material

ESM 1 (DOCX 29 kb)
