# Supplementary material for: Selecting the dosage of ceftazidime–avibactam in the perfect storm of nosocomial pneumonia
Source: Eur J Clin Pharmacol. 2019 Dec 14;76(3):349–61. doi: 10.1007/s00228-019-02804-z (PMC7223046; doi:10.1007/s00228-019-02804-z)
Supplement: Supplementary file 1 — (DOCX 29 kb) [file 228_2019_2804_MOESM1_ESM.docx]

# Supplementary materials

## Limitations of bronchoalveolar lavage sampling and epithelial lining fluid concentration measurements

Bronchoalveolar lavage procedures are invasive, which makes it difficult to guarantee that samples will be collected from every patient and it is impossible to collect repeated samples from the same patient over a time course [1]. In addition, the standard urea dilution method used to estimate epithelial lining fluid (ELF) dilution is prone to errors in sample collection and processing [2]. Performing such a test on all patients enrolled in a clinical trial would be both impractical and unethical, so, for ceftazidime-avibactam it was necessary to extrapolate ELF exposures in infected patients from data in healthy subjects and a murien model using plasma exposures. Serial plasma samples can be obtained easily from patients and the methodology for processing plasma samples is more robust than measurement of concentrations in ELF obtained from BAL samples. For ceftazidime and avibactam, the plasma PK profiles are well characterized, as both drugs are administered intravenously and have short half-life disposition kinetics with mainly renal elimination [3].

## In vitro interaction with other antibiotics

Another potential confounder of antimicrobial activity is the presence of other antibiotics; this is particularly important for serious infections, such as nosocomial pneumonia (NP), for which multiple antibiotic therapy is common [4, 5]. Dallow and colleagues reported that no antagonism was observed in vitro between ceftazidime-avibactam and six different antimicrobial agents of different classes (tobramycin, levofloxacin, linezolid, vancomycin, tigecycline, and colistin) when tested against aerobic bacterial species frequently isolated from patients with NP [6].

## Parameter estimates for the final ceftazidime population PK model

| **Parameter (units)** | **Estimate** | **%RSE** | **BSV (CV%)** |
| --- | --- | --- | --- |
| Slope 1: CL_CR_ <100 mL/min, slope1*CL_CR_ | 0.0103036 | 0.409 | NA |
| Slope 2: CL_CR_ ≥100 mL/min, slope1*100 + slope2*(CrCLCrCLCrCLCL_CR_–100) | 0.001252 | 8.84 | NA |
| θ_1_: CL (L/h) | 6.95 | 1.7 | 42.3 |
| θ_2_: *V*_c_ (L) | 10.5 | 13.1 | 105 |
| θ_3_: Q (L/h) | 31.5 | 18.8 | 259 |
| θ_4_: *V*_p_ (L) | 7.57 | 9 | 110 |
| θ_5_: Population effect on CL (cIAI) | 1.16 | 2.2 | NA |
| θ_6_: Population effect on CL (NP) | 0.999 | 2.4 | NA |
| θ_7_: Race effect on CL (ASN) | −0.161 | 11.8 | NA |
| θ_8_: Race effect on CL | −0.0855 | 27 | NA |
| θ_9_: Population effect on *V*_c_ (cUTI) | 1.03 | 11.1 | NA |
| θ_10_: Population effect on *V*_c_ (cIAI or NP) | 1.14 | 9.9 | NA |
| θ_11_: Population effect on *V*_c_ (cUTI/acute pyelonephritis) | −0.185 | 41.2 | NA |
| θ_12_: Race effect on *V*_c_ (ASN, CHN, JPN) | −0.27 | 18.6 | NA |
| θ_13_: WT effect on *V*_c_ | 1.01 | 12.6 | NA |
| θ_14_: Population effect on *V*_c_ (NPv) | 0.297 | 45.4 | NA |
| Shrinkage (%) or correlation^a^ | | | |
| ηCL^2^ | 0.179 | 3.3 | 11.4 |
| ηV_c_^2^ | 1.10 | 10.2 | 31.2 |
| η*V*_c_–ηCL covariance^b^ | −0.189 | 15.2 | *r* = −0.42 |
| η*V*_p_^2^ | 1.21 | 8.8 | 17.5 |
| η*V*_p_–ηCL covariance^b^ | 0.383 | 5.1 | *r* = 0.82 |
| η*V*_p_–η*V*_c_ covariance^b^ | −0.972 | 7.3 | *r* = −0.84 |
| ηQ^2^ | 6.70 | 15.5 | 27.46 |
| ηQ–ηCL covariance^b^ | 0.883 | 10.1 | *r* = 0.81 |
| ηQ–η*V*_c_ covariance^b^ | −0.643 | 43.1 | *r* = −0.24 |
| ηQ–η*V*_p_ covariance^b^ | 1.73 | 14.5 | *r* = 0.61 |
| Residual noise | | | |
| Proportional error, phase I^b^ | 0.04 | 0.5 | 2.9 |
| Additive error, phase I^b^ | 26489 | 7.5 | 2.9 |
| Proportional error, phase II and phase III^b^ | 0.114 | 2.1 | 9.5 |
| Additive error, phase II and phase III^b^ | 18.4 | 447 | 9.5 |

*ASN* non‐Japanese, non‐Chinese Asian, *BSV* between‐subject variability, *CHN* Chinese, *cIAI* complicated intra‐abdominal infection, *CL* clearance *CL_CR_* creatinine clearance, *cUTI* complicated urinary tract infection, *CV%* coefficient of variation, *JPN* Japanese, *η* individual random subject effect, *NA* not assessed, *NP* nosocomial pneumonia, *NPv* NP with the presence of a ventilator in the hospital room on the day of PK sampling, which includes ventilator‐associated pneumonia and hospital‐acquired pneumonia in patients who were receiving ventilation on the day of sampling, *θ* typical value of PK parameter, *Q* intercompartmental clearance, *%RSE* % relative standard error, *V*_c_ volume of the central compartment, *V*_p_ volume of the peripheral compartment, *WT* body weight

^a^ Correlation coefficient (*r*) between random effects

^b^ Reported as variance

Data from Li et al. 2019 [7]

## Parameter estimates for the final avibactam population PK model

| **Parameter (units)** | **Estimate** | **%RSE** | **BSV (CV%)** |
| --- | --- | --- | --- |
| θ_1_: CL (L/h) | 10.2 | 1.8 | 59.1 |
| θ_2_: *V*_c_ (L) | 11.1 | 9.9 | 107.1 |
| θ_3_: Q (L/h) | 5.44 | 13.9 | 122.2 |
| θ_4_: *V*_p_ (L) | 6.91 | 6.5 | 252.2 |
| θ_5_: CL estimate for patients with ESRD | 0.0678 | 8.3 | NA |
| θ_6_: CL estimate for patients on dialysis | 20.8 | 9.6 | NA |
| θ_7_: Power CL_CR_ (<80) on CL | 1.05 | 2.4 | NA |
| θ_8_: Linear CL_CR_ (≥80) on CL | 0.00279 | 3.7 | NA |
| θ_9_: Population effect on *V*_c_ (cIAI, phase II), *V*_c_*(1 + θ_9_) | 1.92 | 25.4 | NA |
| θ_10_: Population effect on CL (cIAI, phase II), CL*(1 + θ_10_) | 0.406 | 23.2 | NA |
| θ_11_: Population effect on *V*_c_ (cUTI), *V*_c_*(1 + θ_11_) | 0.434 | 24 | NA |
| θ_12_: Population effect on *V*_c_ (cIAI, phase III, NP), *V*_c_*(1 + θ_11_) | 0.329 | 28.6 | NA |
| θ_13_: Scaling factor for CL_CR_ in subjects with ARC, CL = TVCL*(1 + θ_8_*θ_13_* [CL_CR_–80]) | 0.992 | 17.4 | NA |
| θ_14_: WT on *V*_c_ (WT/70.0)^θ14^ | 1.08 | 7.8 | NA |
| θ_15_: APACHE II on CL, CL*(1 + θ_15_) | −0.197 | 8.7 | NA |
| θ_22_: ASN on CL, CL*(1 + θ_22_) | −0.0865 | 20.2 | NA |
| θ_28_: NPv on *V*_c_,*V*_c_*(1 + θ_28_) | 0.175 | 53.3 | NA |
| Shrinkage (%)^a^ | | | |
| ηCL^2^ | 0.349 | 2 | 7.29 |
| η*V*_c_ ^2^ | 1.147 | 6 | 28.15 |
| η*V*_c_–ηCL^b^ | 0.125 | 15.6 | *r* = 0.2 |
| η*V*_p_^2^ | 1.494 | 7 | 13.52 |
| η*V*_p_–ηCL^b^ | 0.611 | 3.6 | *r* = 0.85 |
| η*V*_p_–η*V*_c_^b^ | −0.426 | 18 | *r* = −0.33 |
| ηQ^2^ | 6.359 | 8.1 | 14.18 |
| ηQ–ηCL^b^ | 1.231 | 4.1 | *r* = 0.83 |
| ηQ–η*V*_c_^b^ | −0.978 | 16.8 | *r* = −0.36 |
| ηQ–η*V*_p_^b^ | 3.059 | 7.1 | *r* = 0.99 |
| Residual noise | | | |
| θ_17_: Proportional error, phase I | 0.173 | 0.1 | NA |
| θ_18_: Additive variability, phase I | 44.6 | 0.5 | NA |
| θ_19_: Proportional variability, phase II | 0.492 | 3 | NA |
| θ_20_: Proportional variability, phase III | 0.363 | 1.1 | NA |

*APACHE II* Acute Physiology and Chronic Health Evaluation II, *ARC* augmented renal clearance, *ASN* non‐Japanese, non‐Chinese Asian, *BSV* between‐subject variability, *cIAI* complicated intra‐abdominal infection, *CL* clearance; *CL_CR_* creatinine clearance, *cUTI* complicated urinary tract infection, *CV%* coefficient of variation, *ESRD* end‐stage renal disease, *η* individual random subject effect, *NA* not assessed, *NP* nosocomial pneumonia, *NPv* NP with the presence of a ventilator in the hospital room on the day of PK sampling, which includes ventilator‐associated pneumonia and hospital‐acquired pneumonia in patients who were receiving ventilation on the day of sampling, *θ* typical value of PK parameter, *Q* intercompartmental clearance, *%RSE* % relative standard error, *TVCL* typical value of CL, *V_c_* volume of the central compartment, *V_p_* volume of the peripheral compartment, *WT* body weight

^a^ Correlation coefficient (r) between random effects

^b^ Reported as variance

Data from Li et al. 2019 [7]

## Population PK simulations of ceftazidime and avibactam concentration–time courses

The final population PK models for ceftazidime and avibactam were used to simulate concentration–time courses in patients with complicated intra‐abdominal infection (cIAI), complicated urinary tract infection (cUTI) or NP [7]. Simulations incorporated covariate distributions appropriate to each indication (cIAI, cUTI, or NP, including ventilator-associated pneumonia [VAP], non-VAP, and NPv [NP with the presence of a ventilator in the hospital room on the day of PK sampling]) and between-patient variability, but excluded parameter uncertainty and residual variability. To account for the correlation between the ceftazidime and avibactam random effects, the random effects were not simulated parametrically but were bootstrapped from the post hoc values using the following procedure:

1. Each patient’s random effects from the ceftazidime and avibactam models were merged into a single data file in which there was one record for each patient containing all of his/her random effects for both compounds.
2. Patient-level random effect records were bootstrapped within the particular population of interest and read into the simulation model as data columns.

While this approach preserved the inherent correlations, between-subject covariates, and parameter random effects, the potential existed for the between-subject variability to be underestimated because of random effect shrinkage. To ameliorate this risk, post hoc random effect estimates were re-inflated prior to simulation using the following formula:

$${ETA}_{SIM}=\frac{{ETA}_{EST}}{1-\frac{shrink\%}{100}}$$

Where:

- ETA_SIM_ is the re-inflated random effect used in the simulation
- ETA_EST_ is the post hoc ETA as estimated by the final population PK models
- shrink% is the estimated random effect shrinkage expressed as a percentage

## Supplementary references

1. Yamazaki K, Ogura S, Ishizaka A, Oh-hara T, Nishimura M (2003) Bronchoscopic microsampling method for measuring drug concentration in epithelial lining fluid. Am J Respir Crit Care Med 168 (11):1304-1307. doi:10.1164/rccm.200301-111OC
2. Kiem S, Schentag JJ (2008) Interpretation of antibiotic concentration ratios measured in epithelial lining fluid. Antimicrob Agents Chemother 52 (1):24-36. doi:10.1128/aac.00133-06
3. Pfizer (2018) Summary of Product Characteristics: Zavicefta 2 g/0.5 g powder for concentrate for solution for infusion. http://www.ema.europa.eu/docs/en_GB/document_library/EPAR_-_Product_Information/human/004027/WC500210234.pdf
4. Jean SS, Hsueh PR (2011) Current review of antimicrobial treatment of nosocomial pneumonia caused by multidrug-resistant pathogens. Expert Opin Pharmacother 12 (14):2145-2148.doi:10.1517/14656566.2011.599320
5. Tamma PD, Cosgrove SE, Maragakis LL (2012) Combination therapy for treatment of infections with Gram-negative bacteria. Clin Microbiol Rev 25 (3):450-470. doi:10.1128/CMR.05041-11
6. Dallow J, Otterson LG, Huband MD, Krause KM, Nichols WW (2014) Microbiological interaction studies between ceftazidime-avibactam and pulmonary surfactant and between ceftazidime-avibactam and antibacterial agents of other classes. Int J Antimicrob Agents 44 (6):552-556. doi:10.1016/j.ijantimicag.2014.07.023
7. Li J, Lovern M, Green ML, Chiu J, Zhou D, Comisar C, Xiong Y, Hing J, MacPherson M, Wright JG, Riccobene T, Carrothers TJ, Das S (2019) Ceftazidime-avibactam population pharmacokinetic modeling and pharmacodynamic target attainment across adult indications and patient subgroups. Clin Transl Sci 12 (2):151-163. doi:10.1111/cts.12585
